# Surgical nodal sampling established by Commission on Cancer Standard 5.8 is essential for accurate lung cancer staging

**DOI:** 10.1016/j.xjon.2026.101685

**Published:** 2026-02-17

**Authors:** Raheem Bell, Amanda B. Francescatti, Daniel Boffa, Timothy W. Mullett, Matthew A. Facktor, Nirmal K. Veeramachaneni, Ryan C. Jacobs, Frank Schneider, Tina J. Hieken, David D. Odell, Ronald J. Weigel

**Affiliations:** a Cancer Programs, American College of Surgeons, Chicago, Ill; b Department of Surgery, Northwestern University, Chicago, Ill; c Division of Thoracic Surgery, Department of Surgery, Yale University School of Medicine, New Haven, Conn; d Markey Cancer Center, Lexington, Ky; e Department of Thoracic Surgery, Geisinger Heart & Vascular Institute, Danville, Pa; f Department of Surgery, St Louis University, St Louis, Mo; g Department of Pathology and Laboratory Medicine, Emory University School of Medicine, Atlanta, Ga; h Division of Breast and Melanoma Surgical Oncology, Department of Surgery, Mayo Clinic, Rochester, Minn; i Department of Surgery, Section of Thoracic Surgery, University of Michigan Medical School, Ann Arbor, Mich; j Department of Surgery, University of Iowa, Iowa City, Iowa

**Keywords:** mediastinal, staging, sampling, non–small cell lung cancer

## Abstract

**Objective:**

Accurate mediastinal staging is critical for the effective treatment of non–small cell lung cancer because lymph node involvement significantly influences prognosis and therapeutic decisions. We sought to evaluate the diagnostic accuracy, limitations, and complementary roles of endobronchial ultrasound-guided transbronchial needle aspiration (EBUS-TBNA), cervical mediastinoscopy, and surgical lymph node sampling in mediastinal staging of non–small cell lung cancer.

**Methods:**

A systematized literature review was performed using PubMed and national guideline repositories. Studies were included if they reported or provided sufficient data to calculate the negative predictive value (NPV) for EBUS-TBNA, mediastinoscopy, or surgical lymph node sampling. Data were synthesized qualitatively across different clinical scenarios.

**Results:**

The pooled (unweighted) NPV of EBUS-TBNA was 93.2% (range, 84.7%-98%). Mediastinoscopy demonstrated a pooled NPV of 93.8% (range, 78.8%-97%), with most false negatives attributable to inaccessible stations. Surgical lymph node sampling yielded a pooled NPV of 92.2% (range, 83.6%-96%) for resected nodal stations, although assessment is limited by variability across studies with inconsistent surgical approaches. These data support the need for systematic intraoperative nodal evaluation to confirm pathologic stage and inform treatment selection.

**Conclusions:**

Although EBUS-TBNA is the preferred initial staging modality due to its minimally invasive nature, its diagnostic limitations warrant a low threshold for additional nodal evaluation. Systematic intraoperative lymph node evaluation at the time of surgical resection is indispensable for definitive staging, providing clinically actionable data that influences treatment decisions. Optimal staging of non–small cell lung cancer requires a multidisciplinary, individualized approach that combines modalities based on pretest probability, imaging findings, and patient factors.

**Figure:**
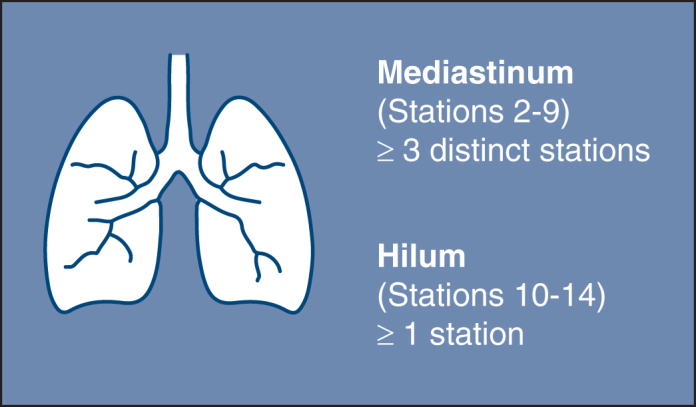
Accurate staging by hilar and mediastinal node sampling.

**Central Message**

Accurate staging in lung cancer requires surgical nodal sampling of ≥1 hilar and ≥3 mediastinal nodes during curative intent pulmonary resection, as described in Commission on Cancer Standard 5.8.

**Perspective**

Accurate mediastinal staging is critical for the effective treatment of lung cancer. Although minimally invasive nodal sampling by endobronchial ultrasound-guided; transbronchial needle aspiration is appropriate for initial evaluation, a negative result requires surgical nodal sampling at the time of curative intent pulmonary resection, as described in Commission on Cancer Standard 5.8.

Lung cancer persists as the leading cause of cancer-related mortality.^1^ In 2025, an estimated 226,650 new lung cancer cases were diagnosed in the United States, with 124,730 resultant deaths.^2^ Accurate staging is critical for optimal treatment. Nodal status in non–small cell lung cancer (NSCLC) is a key prognostic and therapeutic determinant.^3^ Upstaging from N0 to N1 to N3 often results in stage progression and prompts a shift from surgery alone to multimodal treatment.^4^ Notably, postsurgical upstaging to pathologic N2 disease occurs in up to 9% to 30% of cases initially classified as clinical N0 or N1.^4^ This underscores the imperative for precise mediastinal staging.

Clinical staging integrates history, exam, imaging, and biopsy to guide initial treatment, but pathologic staging remains the gold standard because it provides definitive assessment. Because of this, the technique and extent of nodal assessment remain the subject of ongoing debate. In response, the American College of Surgeons (ACS) developed Commission on Cancer (CoC) Standard 5.8 to ensure accurate staging for patients undergoing curative-intent lung cancer resection.^5^ This standard mandates sampling of at least 3 distinct mediastinal stations (N2) and 1 hilar station (N1).^5^^,^^6^ This guideline is supported by an observational cohort study showing that sampling at least 3 mediastinal stations improved survival.^7^ Similarly, the National Comprehensive Cancer Network (NCCN) recommends sampling at least 3 distinct N2 lymph nodes, along with intrapulmonary and hilar nodes, based on independent analyses demonstrating that adherence to this standard is associated with improved survival.^8^, ^9^, ^10^

Given the importance of accurate nodal staging in guiding treatment and improving survival, multiple modalities have been developed to assess mediastinal lymph nodes. Endobronchial ultrasound-guided transbronchial needle aspiration (EBUS-TBNA) is a minimally invasive bronchoscopic technique using ultrasound to guide needle aspiration of mediastinal/hilar nodes.^11^ Cervical mediastinoscopy is a surgical procedure requiring general anesthesia, allowing direct biopsy of paratracheal and subcarinal nodes.^11^ Surgical lymph node assessment, via sampling or systematic dissection during curative intent pulmonary resection, is considered the definitive staging method.^12^ This review examines evidence on how these modalities achieve accurate staging; however, there remains debate as to whether a negative preoperative EBUS-TBNA provides adequate assessment for accurate nodal staging.^E1^ This review compares the diagnostic performance and limitations of EBUS-TBNA, mediastinoscopy, and surgical lymph node sampling.

## Materials and Methods

We conducted a systematized literature review to evaluate the diagnostic performance, limitations, and clinical utility of EBUS-TBNA, cervical mediastinoscopy, and surgical lymph node sampling in the mediastinal staging of NSCLC. Institutional review board approval was not required. No patient-specific data was used in the study; all data was based on the published literature.

### Search Strategy

A comprehensive search was performed in PubMed, supplemented by review of clinical guideline repositories (eg, NCCN) and specialty society publications (eg, American College of Chest Physicians and ACS). The search included combinations of keywords including *lung cancer staging*, *mediastinal lymph nodes*, *EBUS versus mediastinoscopy*, *mediastinoscopy accuracy*, *surgical lymph node dissection lung cancer*, *NCCN lung cancer guidelines*, and *Commission on Cancer Standard 5.8*. The search was unrestricted by publication year to incorporate both foundational and contemporary studies. Only English-language studies were included.

### Eligibility Criteria

**Figure 1:**
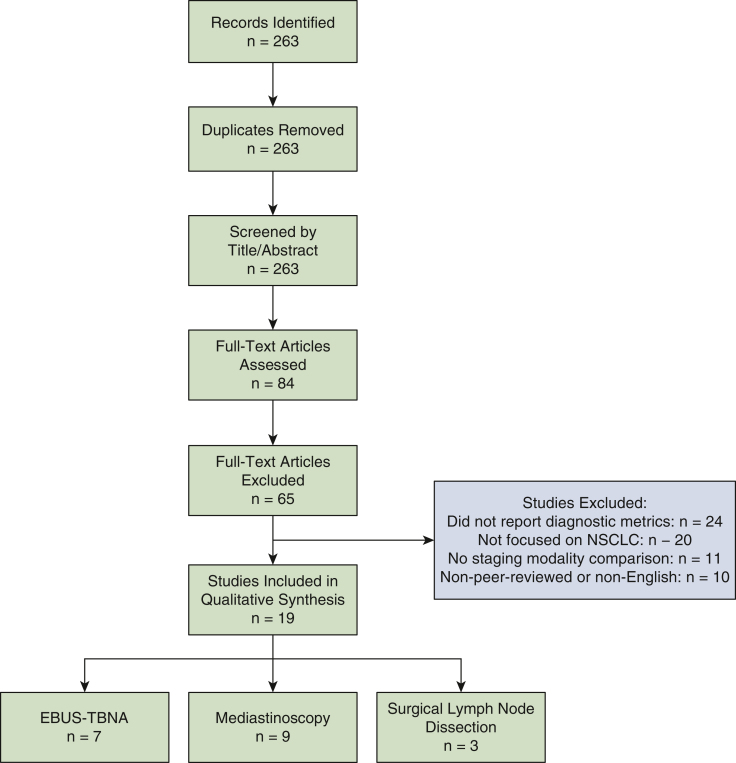
Study Consolidated Standards of Reporting Trials diagram. This figure shows the process employed to identify appropriate articles for inclusion in the analysis. *NSCLC*, Non–small cell lung cancer; *EBUS-TBNA*, endobronchial ultrasound-guided transbronchial needle aspiration.

Included studies met the following criteria: reported diagnostic accuracy metrics (sensitivity, specificity, negative predictive value [NPV]) for EBUS-TBNA, mediastinoscopy, or surgical dissection in NSCLC; provided direct comparisons of staging modalities or evaluated lymph node mapping; discussed technical, procedural, or guideline-based aspects of mediastinal staging; and were peer-reviewed systematic reviews, meta-analyses, randomized controlled trials, observational studies, or evidence-based clinical practice guidelines. Studies were excluded if they focused on cancers other than NSCLC, addressed general thoracic surgery unrelated to nodal staging, and/or lacked extractable diagnostic data. See Figure 1 for details on the process of study inclusion/exclusion.

### Data Extraction and Synthesis

For each eligible source, we extracted the following: study type and population characteristics, number and location of lymph nodes sampled, sensitivity, specificity, and predictive values, and reported procedural risks and limitations. Due to heterogeneity in study designs and reported outcomes, a qualitative synthesis was performed, grouping evidence by modality and clinical scenario. Comparative analyses were conducted to highlight the relative strengths and weaknesses of each staging approach. Key references were cited directly in the results section to support interpretation of diagnostic performance.

## Results

### Diagnostic Performance of EBUS-TBNA

**Figure 2:**
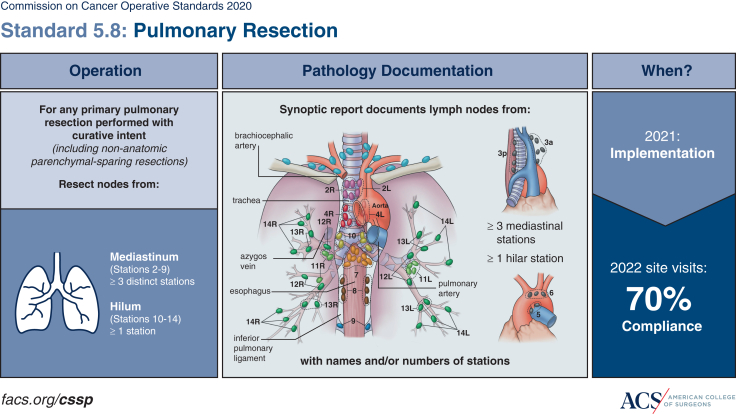
Commission on Cancer (CoC) Operative Standard 5.8: Pulmonary Resection for Curative Intent. This figure outlines the requirements for lymph node dissection in primary pulmonary resections performed with curative intent. *Left*, The figure specifies the minimum number of lymph node stations to be resected: at least 3 distinct mediastinal stations (Stations 2-9) and at least 1 hilar station (Stations 10-14). *Middle*, An anatomical illustration details the common lymph node stations of the lung, indicating that synoptic pathology reports must document lymph nodes from ≥3 mediastinal stations and ≥1 hilar station, including names and/or numbers of stations. *Right*, This standard was implemented in 2021 with a site-level compliance requirement of 70%, as confirmed during 2022 site visits. Reprinted with permission from the American College of Surgeons Cancer Surgery Standards Program Education Committee.

Although EBUS-TBNA has become a primary tool for mediastinal staging because of its minimally invasive nature, it is important to note that not all patients require preoperative nodal assessment. In selected cases where the probability of nodal involvement is likely to be low, such as peripheral tumors ≤2 cm without suspicious nodal disease on computed tomography/positron-emission tomography, clinicians may proceed directly to surgery and perform intraoperative nodal assessment as the initial staging strategy. For patients who do undergo preoperative invasive mediastinal staging, EBUS-TBNA consistently demonstrates high specificity (often ∼100%) for malignancy when adequate aspirates are obtained.^11^^,^^13^ However, the sensitivity of EBUS is more variable. In patients with radiologically normal mediastinum (cN0) or lymph node involvement limited to N1 nodes (hilar or intrapulmonary) (cN1), EBUS-TBNA sensitivity can be lower. Leong and colleagues^4^ reported a pooled sensitivity of 49% (95% CI, 41%-57%) for EBUS-TBNA in patients with cN0/N1 NSCLC. Similarly, Vial and colleagues^14^ and Silvestri and colleagues^15^ found an EBUS-TBNA sensitivity of 40% for unsuspected N2 metastases in early-stage (I or II) NSCLC, with 20% of patients upstaged from clinical N0 or N1 to pathological N2 despite negative EBUS. In contrast, for patients with radiologically-evident nodal involvement (eg, bulky adenopathy), EBUS-TBNA sensitivity can reach 80% to 90%.^11^ Meta-analyses, including mixed-risk populations, report EBUS-TBNA sensitivity around 81% to 89%.^13^^,^^15^ EBUS performance is operator-dependent and prone to sampling error because fine-needle aspiration captures only a cytologic sample.^11^^,^^16^ EBUS provides access to stations 2, 3, 4, 7, and 10 to 13; combined with endoscopic ultrasound (EUS) (accessing stations 3, 5, 7, 8, and 9), it can cover 11 of 14 stations^1^^,^^17^, ^18^, ^19^ (Figure 2).

The utility of EBUS-TBNA hinges on its NPV, which informs how confidently a negative result can exclude mediastinal nodal involvement. A high NPV suggests further invasive staging, such as mediastinoscopy, may be unnecessary. To evaluate this, we reviewed 7 studies (publication years 2013-2024)—including small and large prospective trials as well as meta-analyses—and calculated a pooled (unweighted) NPV of 93.2% (range, 84.7%-98%).^4^^,^^11^^,^^20^, ^21^, ^22^, ^23^, ^24^, ^25^ Notably, the NPV drops significantly to 65.9% when nondiagnostic cases are included.^20^ Additionally, the NPV is lower in patients with cN1 disease compared with cN0, likely reflecting the increased prevalence of occult mediastinal metastases in the cN1 population.^21^

### Diagnostic Performance of Mediastinoscopy

Cervical mediastinoscopy has long been a reference standard. It also exhibits near-perfect specificity (∼100%).^11^^,^^13^ Reported sensitivity ranges from ∼70% to 80%.^11^ One meta-analysis found a median sensitivity of 78% and a NPV of 91%.^11^ In patients with cN1 disease and a negative mediastinum on imaging, 1 study reported mediastinoscopy sensitivity of 73% versus 38% for EBUS in detecting N2 disease.^26^ For clinical N2 NSCLC, mediastinoscopy sensitivity can be very high (95%-97%).^27^ Standard mediastinoscopy accesses stations 2R, 2L, 4R, 4L, and 7.^11^ It is more invasive than EBUS, requiring general anesthesia, with a major complication rate typically <2% to 3%.^11^ We evaluated 9 studies (publication years 2006-2023), including prospective trials and meta-analyses, and calculated a pooled (unweighted) NPV for mediastinoscopy of 93.8% (range, 78.8%-97%).^15^^,^^26^, ^27^, ^28^, ^29^, ^30^, ^31^, ^32^, ^33^ Most of the false negatives during mediastinoscopy were due to stations that were not anatomically accessible (stations 5, 6, 8 and 9).

### Diagnostic Performance of Surgical Lymph Node Sampling

Systematic surgical lymph node sampling during lung resection provides the most definitive pathological staging (pN status).^12^ It approaches 100% sensitivity and specificity for the nodes removed, detecting even microscopic metastases.^11^ The ACS CoC Standard 5.8 mandates thorough intraoperative nodal evaluation, requiring sampling of at least 3 distinct N2 mediastinal stations (including subcarinal station 7) and at least 1 N1 intrapulmonary or hilar node (Figure 1).^5^^,^^34^^,^^35^ This comprehensive approach provides definitive staging for patients who preoperatively are believed to be node-negative and provides definitive staging for patients who proceed directly to curative-intent resection.^5^ However, it is the most invasive approach, reserved for patients undergoing curative intent pulmonary resection.^11^ A pooled analysis of 3 studies (publication years 2002-2011) yielded an NPV of 92.2% (range, 83.6%-96%) for surgical lymph node sampling.^36^, ^37^, ^38^ False negative results of surgical node sampling are attributable to several factors, including poor adherence to lymph node sampling standards, the presence of skip metastases, which are more common in central tumors, difficulty accessing stations 5 and 6 in left upper lobe tumors, and errors in specimen labeling and nodal mapping. The variability in results is influenced by the extent of lymph node sampling because surgery can achieve a very high NPV depending on how thoroughly the dissection is performed.

### Comparative Analysis

Each staging modality has unique strengths and limitations (Table 1). EBUS-TBNA is the least invasive and provides valuable preoperative staging information. It can also be performed under moderate sedation, making it an attractive first-line, preoperative option. However, its diagnostic performance is highly operator-dependent and limited by access to certain nodal stations.^4^ To further complicate the matter, the published sensitivities in EBUS also require the presence of rapid on-site evaluation with cytopathology, which is variable across institutions.^39^ Moreover, adequacy of EBUS-TBNA requires adherence to established protocols that have been established as best practice for the technique.^39^ Although EBUS-TBNA demonstrates near-perfect specificity, its sensitivity varies widely by clinical stage. The pooled NPV of 93.2% (range, 84.7%-98%) suggests that a negative result cannot reliably exclude nodal metastases in all cases, particularly in patients with cN1 disease or nondiagnostic aspirates.

**Table 1:** Diagnostic performance of mediastinal staging modalities

Modality	Pooled NPV∗ (%)	Range (%)	Advantages	Limitations
EBUS-TBNA	93.2	84.7-98	Minimally invasive, good for initial staging, access to multiple nodal stations	Operator-dependent, sampling error, reduced sensitivity in early-stage disease
Mediastinoscopy	93.8	78.8-97	High sensitivity for occult N2 disease, direct visualization, confirmatory after EBUS	Requires general anesthesia, invasive, limited nodal access
Surgical lymph node staging	92.2	83.6-96	Detects microscopic disease, fulfills surgical quality standards	Only applicable intraoperatively, most invasive, not for initial staging

*NPV*, Negative predictive value; *EBUS-TBNA*, endobronchial ultrasound-guided transbronchial needle aspiration.

∗ Although NPV estimates in the literature were derived before the implementation of Commission on Cancer Standard 5.8, emerging data reveal substantial variability in real-world compliance with nodal sampling guidelines. Currently, no studies report NPV specifically for cases that fully adhere to Standard 5.8. Future research should aim to evaluate NPV and other diagnostic performance metrics in this subset and assess the standard's influence on staging accuracy, treatment decisions, and patient survival outcomes.

Mediastinoscopy remains a reference standard for mediastinal staging. It provides direct visualization and allows for more extensive tissue sampling. Its specificity approaches 100%, and sensitivity is generally higher than EBUS-TBNA, particularly for occult N2 disease. The pooled NPV for mediastinoscopy is 93.8% (range, 78.8%-97%), marginally higher than EBUS-TBNA, especially in settings where certain stations (eg, 5, 6, 8 and 9) are not required or expected to be accessed. Of note, this parallels the inability of EBUS-TBNA to access certain stations.

Surgical lymph node sampling has the potential to provide the most comprehensive and definitive staging. It is reserved for patients proceeding to curative intent pulmonary resection and is required by national standards such as CoC Standard 5.8 and NCCN guidelines. A pooled NPV of 92.2% (range, 83.6%-96%) was observed across 3 historical studies, although this performance may be compromised by poor adherence to sampling guidelines, skip metastases, difficulty reaching certain nodal stations, and labeling errors. This NPV reflects the likelihood that patients who had a minimum of 1 N1 and 3 N2 nodal stations sampled surgically and found to be negative likely had N0 disease. False negatives were identified by reviewing complete surgical pathology and determining that some positive nodes had not been sampled or were misclassified, rather than being missed due to disease recurrence^36^^,^^38^^,^^40^^,^^E2^ This was demonstrated in the American College of Surgeons Oncology Group (ACOSOG) Z0030 trial, where positive lymph nodes were found during complete lymphadenectomy in patients who had initially undergone only systematic sampling, revealing that some nodal metastases had been missed by the initial approach.^38^

Surgical nodal evaluation is highly variable, and this variability was 1 of the key motivations for establishing standards such as CoC Standard 5.8. Multiple studies using large national datasets, including the National Cancer Database, have demonstrated that the number of lymph nodes removed during surgery is strongly associated with the likelihood of nodal upstaging. For example, 1 analysis found that patients who had more than 14 lymph nodes removed had a nodal upstaging rate of 17.9%, compared with only 10.9% when fewer nodes were assessed.^E3^ Historically, it was believed that upstaging rates may vary by surgical approach, with open lobectomy consistently showing higher detection of occult nodal disease than video-assisted thoracoscopic surgery or robotic-assisted surgery, even when similar numbers of nodes are examined.^E4^ Contemporarily, it has been well documented than minimally invasive approaches may actually facilitate better nodal dissection. Institutional variation is also considerable, with some high-volume centers averaging fewer than 10 nodes per resection.^E3^ These findings underscore that the quality of surgical nodal staging is far from uniform and reinforce the need for standardized, evidence-based lymph node evaluation practices.

In practice, these modalities are often used sequentially and complementarily. EBUS-TBNA is typically employed first due to its minimally invasive nature, followed by mediastinoscopy if results are negative but clinical suspicion remains high. Surgical nodal sampling confirms nodal status intraoperatively and may provide therapeutic benefit. The choice of modality is guided by clinical context, pretest probability of nodal disease, and available institutional expertise.

The clinical importance of accurate nodal staging goes beyond better prognosis. Nodal upstaging from clinical N0 or N1 to pathologic N2 frequently leads to a shift in treatment strategy from surgery alone to multimodal therapy. For example, improving compliance with CoC Standard 5.8 has been associated with more frequent nodal upstaging in patients meeting the CoC standard (21.3% vs 12.5% when standard not met; *P* = .004).^E5^ This metric can be translated into a tangible survival benefit. In the **Ad**juvant **A**z**u**ra for **R**esected **A**EGFR-mutated NSCLC (ADAURA) trial analysis, adjuvant osimertinib improved the 4-year disease-free survival rate from 38% to 73% in patients with stage IB to IIIA estimated glomerular filtration rate-mutated NSCLC (hazard ratio, 0.27; 95% CI, 0.21-0.34).^E6^ This substantial benefit highlights the importance of accurate staging, as even a 5% increase in nodal upstaging could expand access to life-prolonging adjuvant therapies. Nodal upstaging is a clinical signal that triggers appropriate treatment.

## Discussion

### Integrating Staging Modalities in Clinical Practice

The optimal mediastinal staging strategy balances diagnostic thoroughness with patient safety and resource use. A stepped approach, guided by pretest probability, is common. In carefully selected low-risk early-stage NSCLC, a negative EBUS-TBNA may be acceptable, but selection should be guided by tumor size, location, imaging findings, and access to relevant nodal stations.^11^ A positive EBUS-TBNA confirms advanced disease, often obviating further surgical staging and guiding patients toward neoadjuvant or definitive chemoradiation.^11^ However, when EBUS-TBNA is negative in a patient with suspicious nodes or high-risk features, confirmatory mediastinoscopy is often indispensable.^1^^,^^11^ NCCN and other guidelines emphasize this confirmatory role if clinical suspicion persists. Combining EBUS with EUS can enhance the diagnostic yield of minimally invasive staging.^E7^^,^^E8^ However, relying solely on EBUS-TBNA may miss occult nodal disease in select patients. A recent meta-analysis by Sanz-Santos and colleagues^23^ reviewed outcomes of confirmatory mediastinoscopy following negative EBUS and found that mediastinoscopy increased the NPV from 79.2% for EBUS-TBNA alone to 91.8% for EBUS-TBNA plus mediastinoscopy. These findings support a stepped approach to staging, where confirmatory mediastinoscopy remains essential in patients with persistent clinical or radiographic suspicion of nodal involvement despite a negative EBUS result.^23^ Although it is critical to take into account the potential for definitive treatment delay, and in turn, potential disease progress while utilizing this stepped approach. A single anesthetic approach that incorporates staging and treatment may be ideal for patient outcomes.

We now have operative standards for lung cancer that are both current and evidence-based, demonstrating improvement in both staging accuracy and patient outcomes. As a result, these standards have been endorsed by major cancer governing bodies in the United States, including the NCCN and the ACS. The rationale is clear: complete resection of lymph nodes provides the most accurate staging information, allowing clinicians to determine the need for adjuvant therapy, which has been shown to improve survival. Rather than re-establishing the diagnostic performance of individual staging modalities, this review contextualizes these data within contemporary surgical quality standards and highlights why preoperative staging alone does not obviate the need for systematic intraoperative nodal evaluation as required by CoC Standard 5.8.

Although EBUS-TBNA can perform on par with surgical staging in optimal conditions and may even outperform it in select cases, there are important limitations. However, its performance varies depending on the lymph node station involved, with poorer results in central tumors and tumors located on the left side.^21^ EBUS also has a reduced ability to detect micrometastases. One study found that 6.8% of EBUS-TBNA specimens missed micrometastases, which was associated with significantly worse progression-free survival (median 210 days compared with 1293 days) and overall survival (239 days compared with 1120 days).^E9^ Missed micrometastases was found at the time of surgical resection. These findings suggest that undetected micrometastatic disease may reflect a more aggressive tumor biology, but also if these patients in reality had more advanced disease to begin with, then this would be a missed therapeutic opportunity for neoadjuvant therapy. Sample adequacy is also a concern, with limitations arising from internal necrosis of lymph nodes, insufficient cellular material, and technical sampling difficulties.^20^^,^^E10^^,^^E11^

Clinical decision making should incorporate the clinical stage, risk factors for occult metastases, the accessibility of nodal stations by EBUS, the quality of the specimen obtained, consideration for timely care delivery, the patient's fitness for additional procedures, and their overall goals of care. It is also important to recognize that many studies report diagnostic accuracy only for specimens that are deemed adequate by the pathologist. If nondiagnostic samples are included in the analysis, the true NPV of EBUS may be much lower than reported. Additionally, studies reporting NPV for EBUS-TBNA tended to have a lower prevalence of occult N2 or N3 disease, which likely overestimated NPV (Table 2, Table 3, Table 4).

**Table 2:** Prevalence of N2 or N3 mediastinal disease in the endobronchial ultrasound cohort

First author, year	Prevalence of N2 or N3 disease (%)	NPV (%)	Sample size
Guinde J, 2020^21^	11.8	94	238
Serra Mitjà P, 2024^22^	5.1	98	118
Sanz-Santos J, 2022^23^	20	91.8	meta
Ong P, 2015^24^	7.7	84.7	220
Shingyoji M, 2014^25^	17.6	90.2	113
Leong TL, 2019^4^	15	94	1146
Whitson BA, 2013^20^	5.8	98	120
Mean	11.8	93.2∗	

*NPV*, Negative predictive value.

∗ Pooled.

**Table 3:** Prevalence of N2 or N3 mediastinal disease in the mediastinoscopy cohort

First author, year	Prevalence of N2 or N3 disease (%)	NPV (%)	Sample size
İskender I, 2011^29^	25	92.5	196
Decaluwe H, 2020^26^	26	92	105
Xiao R, 2019^27^	70	92.3	87
Lemaire A, 2006^28^	23.5	94.5	1459
Diebels I, 2020^30^	45.2	91.9	168
Silva JS, 2019^31^	19.2	93.2	213
Majeed FA, 2023^32^	45	97	60
Um SW, 2015^33^	59.1	78.8	127
Silvestri GA, 2013^15^	20-60	91	2070
Mean	39.1	93.8∗	

*NPV*, Negative predictive value.

∗ Pooled.

**Table 4:** Prevalence of N2 or N3 mediastinal disease in the surgical lymph node sampling cohort

First author, year	Prevalence of N2 or N3 disease (%)	NPV (%)	Sample size
Wu N, 2011^36^	23.6	90.3	110
Massard G, 2006^37^	28.8	83.6	208
Darling GE, 2011^38^	4	96	525
Mean	18.8	92.2∗	

*NPV*, Negative predictive value.

∗ Pooled.

Patients with clinical N1 disease are at higher risk for false-negative results with EBUS-TBNA, likely due to a greater prevalence of occult mediastinal metastases and limitations in nodal access.^21^ This raises an important consideration as to whether EBUS-TBNA should be limited to patients with radiographic cN0 disease. Pursuing multiple staging approaches for more radiological advanced tumors may lead to delays in care secondary to repeated testing, and risk tumor progression and patient outcomes. Furthermore, as with operative standards for surgical lymph node staging, there is a need to establish evidence-based criteria for when EBUS alone may be sufficient. Optimal candidates for EBUS-only staging are likely those with peripheral tumors <2 cm in size, located in the lower lobes, and without suspicious lymphadenopathy on imaging. In these cases, particular attention should be paid to stations 5 and 6, which are not routinely accessible via EBUS, as well as stations 8 and 9, which require EUS and may be involved in lower lobe disease.^E12^

### Emerging Clinical Scenarios That Complicate Mediastinal Staging

Contemporary lung cancer staging increasingly serves a dual purpose: defining anatomic disease extent while ensuring adequate tissue acquisition to support biomolecular profiling and multimodality treatment selection. Although needle-based techniques such as EBUS-TBNA have traditionally emphasized cytologic diagnosis, emerging approaches including EBUS-guided cryobiopsy may enhance tissue adequacy for molecular analyses.^1^ CoC Standard 5.8 is agnostic to the preoperative staging technique used and instead emphasizes systematic nodal evaluation, which remains essential regardless of how initial tissue was obtained.

Mediastinal staging is further complicated in patients with prior nodal sampling or dissection, including those with multifocal ipsilateral or bilateral primaries. Previous TBNA or lymphadenectomy may alter nodal architecture through fibrosis or inflammation, potentially reducing the accuracy of subsequent staging efforts.^15^ In these scenarios, systematic intraoperative nodal evaluation at the time of definitive resection remains critical for accurate pathologic staging.

Among surgeons, uncertainty persists regarding whether the CoC 3 + 1 requirement implies nodal sampling versus complete mediastinal lymph node dissection. Standard 5.8 specifies systematic sampling of designated nodal stations rather than formal mediastinal lymphadenectomy; however, sampling is intended to remove representative nodal tissue from each station rather than selective node cherry picking. Variability in interpretation and execution likely contributes to heterogeneity in staging quality.^5^^,^^8^^,^^28^

Finally, the expanding use of neoadjuvant immunotherapy introduces additional complexity due to treatment-related nodal inflammation and pseudoprogression. Positron-emission tomography-positive mediastinal nodes following induction therapy may not reliably distinguish residual malignancy from immune response. In this setting, surgical nodal evaluation at the time of resection remains the reference standard for definitive pathologic staging and treatment allocation.^E13^

### Limitations of Needle-based Staging and the Ongoing Role of Mediastinoscopy

Although EBUS-TBNA has become a widely used minimally invasive staging tool, needle-based approaches come with important limitations. False negatives can occur due to sampling error, particularly when only a limited number of nodal stations are assessed or when metastatic disease is focal.^11^^,^^16^ The **A**ssessment of **S**urgical S**t**aging versus **E**ndosonographic Ultrasound in Lung Cancer: A **R**andomised Clinical Trial (ASTER) suggested that combining EBUS and EUS could reduce the need for surgical staging; however, the trial had key limitations. The median number of stations sampled was only 3 with EBUS/EUS compared with 4 with mediastinoscopy, and the trial excluded patients with cN0 disease, limiting its generalizability.^11^^,^^E14^ Nondiagnostic samples, typically defined as specimens lacking sufficient lymphocytes or cellularity for interpretation, are observed in approximately 9% to 13% of EBUS-TBNA cases.^15^^,^^18^^,^^20^ Rates vary depending on operator experience and lymph node characteristics. These samples are often excluded from accuracy calculations, which may lead to an overestimation of EBUS performance. Additionally, most EBUS operators are not trained in EUS, which may further reduce the accuracy widely.

Operator experience significantly influences the diagnostic performance of EBUS. Although EBUS-TBNA is widely used for mediastinal staging, its accuracy is influenced by sampling technique, nodal station accessibility, and specimen adequacy. Although mediastinoscopy offers direct visualization and confirmation of sampling, its use has declined over time. Complications from mediastinoscopy are rare, but when they do occur, they can be severe and may influence decision making and contribute to a reduction in the utilization of this approach. Among thoracic surgery operations, mediastinoscopy was performed in 14.6% of cases in 2006 but only 11.4% by 2010, with median center rates dropping from 21.4% to 10.0% over the same period.^E15^ Despite this trend, mediastinoscopy may still play a role in select cases, such as restaging after induction therapy, where fibrosis can limit the effectiveness of needle aspiration.^11^

### Implications for Surgical Practice and Nodal Sampling Standards

Accurate staging improves patient management, and the opportunity to positively influence prognosis, underscoring the importance of increased emphasis on surgical quality measures like CoC Standard 5.8.^5^^,^^11^ This standard mandates systematic lymph node evaluation during curative-intent resections (at least 1 intrapulmonary or hilar station and 3 distinct mediastinal stations, including station 7).^5^^,^^6^ The importance of systematic nodal evaluation is further reinforced by the concept of R-uncertain resection, a classification in which inadequate nodal assessment is a dominant driver and is associated with worse survival despite ostensibly complete resection.^E16^^,^^E17^ Adherence minimizes understaging and ensures patients receive appropriate adjuvant therapy if occult N2 disease is found. This highlights that, for patients undergoing curative-intent lung cancer resection, a systematic approach to lymph node sampling can lead to better long-term outcomes by informing correct postoperative treatment. Preoperative staging guides appropriate decision making regarding surgical candidacy, whereas intraoperative dissection provides final pathologic confirmation of stage and informs adjuvant treatment allocation. However, emerging evidence suggests that systematic lymph node sampling may be safely omitted in highly selected patients. A recent Phase 3 randomized trial from high-volume centers in East Asia found no nodal metastases among patients with ground-glass opacity dominant lung adenocarcinoma and reported fewer complications and shorter recovery when mediastinal lymphadenopathy was omitted.^E18^ Although this population is far more prevalent in Asian centers and is not as commonly encountered in US practice, and current American guidelines do not directly address ground-glass opacity-dominant tumors, the study highlights the potential for more personalized staging strategies based on individualized risk. These findings underscore that the quality of surgical nodal staging is far from uniform and reinforce the need for standardized, evidence-based lymph node evaluation practices. Building on this, mediastinal staging decisions are most effective when made within a multidisciplinary framework that integrates imaging, biopsy results, comorbidities, and patient preferences while aligning with NCCN and ACS guidelines.

### Limitations

There are several limitations in this review. The selection of studies, although aiming for comprehensiveness, may be subject to selection bias, and not all available data, particularly from smaller or non-English language studies, may have been excluded. The field is rapidly evolving, and new technologies or refinements to existing techniques could alter the current comparative landscape. Operator and institutional variability in performing EBUS-TBNA and mediastinoscopy can influence reported outcomes, and aggregated data may not reflect performance in all settings. This review primarily focused on diagnostic accuracy; patient-centered factors like quality of life, comorbidities, and preferences, which significantly influence clinical decision making, were not explored in depth. Furthermore, a formal analysis of downstream outcomes like long-term survival based on specific staging strategies was beyond the scope of this study. One of the real challenges in determining the effectiveness of clinical staging evaluations is the ability to know true disease status—more specifically, how to tell the patient's true extent of nodal involvement to benchmark performance of clinical evaluations. Currently, surgical nodal sampling is relied upon as the gold standard, but if this is not performed appropriately, patients may be inaccurately understaged.

## Conclusions

Accurate mediastinal staging in NSCLC is critical for guiding therapy and prognostication. This comprehensive review underscores that although minimally invasive techniques like EBUS-TBNA (with or without EUS) have revolutionized initial staging, their inherent limitations, particularly in sensitivity and the potential for sampling error, must be recognized. Needle-based staging offers high specificity but a negative result, especially in high-risk scenarios or when sampling is incomplete, does not definitively exclude nodal involvement.

Cervical mediastinoscopy maintains a crucial complementary role, providing a higher degree of diagnostic certainty when needle biopsies are negative despite persistent clinical suspicion, or in specific restaging contexts. Surgical lymph node sampling, performed systematically during curative-intent resection as advocated by CoC Standard 5.8, remains the definitive standard for intraoperative lymph node staging. This ensures the most precise stage determination, which is vital for guiding adjuvant therapies and optimizing long-term outcomes. In clinical practice, an individualized, evidence-based, and multidisciplinary approach is paramount. EBUS-TBNA is appropriately utilized as the initial preoperative staging method for most patients with operable NSCLC. However, a low threshold for confirmatory mediastinoscopy should be maintained in cases of negative EBUS with high pretest probability of mediastinal disease where the decision for surgery might be influenced. For all patients undergoing curative intent surgical resection, adherence to systematic lymph node dissection protocols, as described by the CoC Standard 5.8, should be performed. By strategically leveraging the strengths of each available modality, clinicians can navigate the complexities of mediastinal assessment, minimizing understaging while avoiding unnecessary invasiveness, ultimately leading to the best possible outcomes for patients with lung cancer.

## Conflict of Interest Statement

Drs Bell, Francescatti, and Weigel hold leadership or advisory roles within the American College of Surgeons Commission on Cancer. All other authors reported no conflicts of interest.

The *Journal* policy requires editors and reviewers to disclose conflicts of interest and to decline handling or reviewing manuscripts for which they may have a conflict of interest. The editors and reviewers of this article have no conflicts of interest.
